# Contributions of connectional pathways to shaping Alzheimer’s disease pathologies

**DOI:** 10.1093/braincomms/fcae459

**Published:** 2025-01-06

**Authors:** Salma Bougacha, Daniel Roquet, Brigitte Landeau, Elise Saul, Mikaël Naveau, Siya Sherif, Alexandre Bejanin, Marc Dhenain, Ashish Raj, Denis Vivien, Gaël Chetelat

**Affiliations:** Normandie Univ, UNICAEN, INSERM, U1237, PhIND ‘Physiopathology and Imaging of Neurological Disorders’, Institut Blood and Brain @ Caen-Normandie, Cyceron, 14000 Caen, France; Normandie Univ, UNICAEN, INSERM, U1237, PhIND ‘Physiopathology and Imaging of Neurological Disorders’, Institut Blood and Brain @ Caen-Normandie, Cyceron, 14000 Caen, France; Normandie Univ, UNICAEN, INSERM, U1237, PhIND ‘Physiopathology and Imaging of Neurological Disorders’, Institut Blood and Brain @ Caen-Normandie, Cyceron, 14000 Caen, France; Normandie Univ, UNICAEN, INSERM, U1237, PhIND ‘Physiopathology and Imaging of Neurological Disorders’, Institut Blood and Brain @ Caen-Normandie, Cyceron, 14000 Caen, France; Normandie Univ, UNICAEN, CNRS, UMS 3408, Cyceron, 14000 Caen, France; Normandie Univ, UNICAEN, INSERM, U1237, PhIND ‘Physiopathology and Imaging of Neurological Disorders’, Institut Blood and Brain @ Caen-Normandie, Cyceron, 14000 Caen, France; Normandie Univ, UNICAEN, INSERM, U1237, PhIND ‘Physiopathology and Imaging of Neurological Disorders’, Institut Blood and Brain @ Caen-Normandie, Cyceron, 14000 Caen, France; Sant Pau Memory Unit, Department of Neurology, Hospital de la Santa Creu i Sant Pau, Biomedical Research Institute Sant Pau, Universitat Autònoma de Barcelona, 08025 Barcelona, Spain; Center of Biomedical Investigation Network for Neurodegenerative Diseases (CIBERNED), 28031 Madrid, Spain; Université Paris-Saclay, CEA, CNRS, Laboratoire des Maladies Neurodégénératives: mécanismes, thérapies, imagerie, F-92265 Fontenay-aux-Roses, France; Commissariat à l’Energie Atomique et aux Energies Alternatives (CEA), Direction de la Recherche Fondamentale (DRF), Institut François Jacob, MIRCen, F-92265 Fontenay-aux-Roses, France; Department of Radiology and Biomedical Imaging, University of California, San Francisco, CA 94143-0628, USA; Normandie Univ, UNICAEN, INSERM, U1237, PhIND ‘Physiopathology and Imaging of Neurological Disorders’, Institut Blood and Brain @ Caen-Normandie, Cyceron, 14000 Caen, France; Département de Recherche Clinique, CHU Caen-Normandie, 14033 Caen, France; Normandie Univ, UNICAEN, INSERM, U1237, PhIND ‘Physiopathology and Imaging of Neurological Disorders’, Institut Blood and Brain @ Caen-Normandie, Cyceron, 14000 Caen, France

**Keywords:** Alzheimer’s disease, pathology spreading, structural connectivity, functional connectivity, relative importance analysis

## Abstract

Four important imaging biomarkers of Alzheimer’s disease, namely grey matter atrophy, glucose hypometabolism and amyloid-β and tau deposition, follow stereotypical spatial distributions shaped by the brain network of structural and functional connections. In this case-control study, we combined several predictors reflecting various possible mechanisms of spreading through structural and functional pathways to predict the topography of the four biomarkers in amyloid-positive patients while controlling for the effect of spatial distance along the cortex. For each biomarker, we quantified the relative contribution of each predictor to the variance explained by the model. We also compared the contribution between apolipoprotein E-ɛ4 carriers and non-carriers. We found that topological proximity to areas of maximal pathology through the functional connectome explained significant parts of variance for all biomarkers and that functional pathways totalized more than 30% of contributions for hypometabolism and amyloid load. By contrast, atrophy and tau load were mainly predicted by structural pathways, with major contributions from inter-regional diffusion. The ɛ4 allele modulated contributions to the four biomarkers in a way consistent with compromised brain connectomics in carriers. Our approach can be used to assess the contribution of concurrent mechanisms in other neurodegenerative diseases and the possible modifying impact of relevant factors on this contribution.

## Teaser

The relative importance of different connectional pathways in shaping Alzheimer’s disease pathologies is unknown. Bougacha *et al.* reported that functional and structural connectome differently contribute to the spreading of grey matter atrophy, glucose hypometabolism and amyloid-β and tau deposition spread in apolipoprotein E-ɛ4 carrier and non-carrier patients with Alzheimer’s disease.

## Graphical Abstract

**Graphical Abstract:**
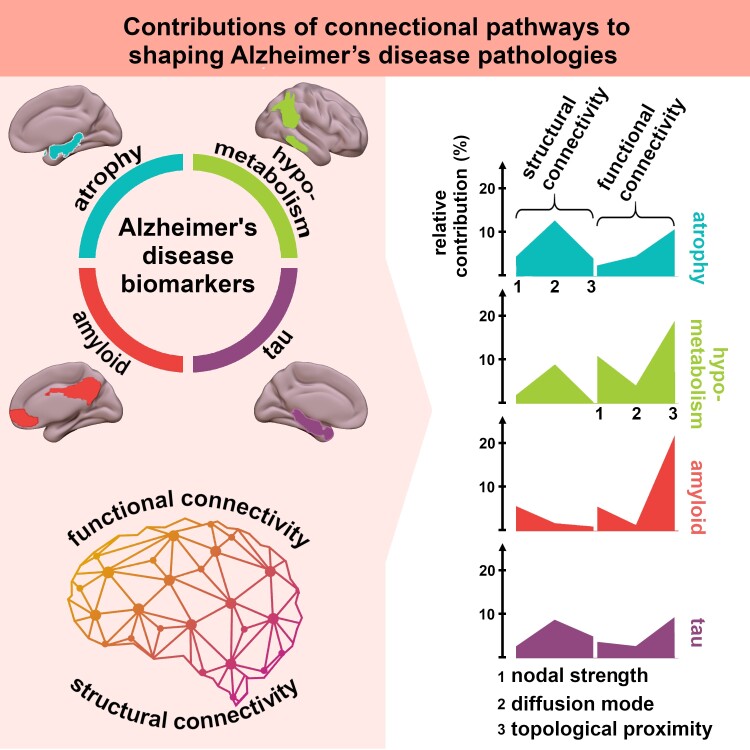

## Introduction

Neuronal connections have long been suspected to subserve the spread of pathology from one brain area to remote areas in many neurodegenerative diseases, including Alzheimer’s disease.^1^ Combining molecular imaging for *in vivo* assessment of Alzheimer’s disease pathology to functional MRI, studies revealed that collections of widespread brain regions communicating together in healthy subjects mirror the stereotyped topography of grey matter atrophy,^2^ glucose hypometabolism,^3^ amyloid-β deposition^4^ and tau deposition,^5^ in Alzheimer’s disease. Major white matter fibre bundles reconstructed in the living human brain thanks to diffusion MRI tractography were also found to be involved in the progression of Alzheimer’s disease.^6-9^

Yet crucial aspects of the inter-regional spread of Alzheimer’s disease pathology remain to be elucidated. The mechanisms of disease progression through the structural and functional macroscale networks are not fully understood. Moreover, the extent to which the involvement of functional pathways reflects genuine coupling between pathological and functional processes, spread along white matter tracts, or the confounding effect of spatial distances between cortical regions, is unknown. We sought to quantify how much each of the major mechanisms proposed in the literature, whether taking place on structural or functional pathways, contributes to shaping the topography of Alzheimer’s disease pathologies independently of spatial distance along the cortex. We focused on the following mechanisms: (i) nodal stress, where highly connected nodes—the network hubs—experience more damage^10-13^; (ii) diffusion of disease effects proportionally to the weight of the outgoing connections^7,14,15^ and (iii) propagation of pathology throughout the brain.^6,13,16-18^ We also investigated the influence of apolipoprotein E (APOE)-ɛ4 carriage, the main genetic factor for Alzheimer’s disease, on these contributions.

## Materials and methods

### Data description

#### Alzheimer’s disease patients and matched controls

This study used T_1_-weighted MRI, ^18^F-fluorodeoxyglucose (^18^F-FDG) PET, ^18^F-florbetapir amyloid-PET and ^18^F-flortaucipir tau-PET images from the Alzheimer’s Disease Neuroimaging Initiative (ADNI) database.^19^ All participants signed written informed consent for participation in the ADNI, as approved by the institutional board at each participating center. Only subjects who had 3T T_1_-weighted MRI, ^18^F-FDG-PET and ^18^F-florbetapir PET scans separated by at most 90 days were considered for atrophy, hypometabolism and amyloid-β deposition. Demographics, clinical assessments and global ^18^F-florbetapir PET standardized uptake value ratio were also downloaded from the ADNI database. Subjects were categorized as amyloid-positive or negative by comparing their global standardized uptake value ratio to the previously established threshold (1.11).^20,21^ Preprocessed images were visually inspected, and participants with corrupted T_1_-weighted or PET images or failed cortical reconstruction were discarded. The whole selection process resulted in the inclusion of 353 amyloid-positive patients (195 females) aged between 55 and 90 years (mean age 73.5) that had a clinical diagnosis of mild cognitive impairment (MCI) or Alzheimer’s disease and 147 age-, sex- and education-matched amyloid-negative cognitively unimpaired participants (65 females) aged between 60 and 96 years (mean age 74). Due to the relatively small number of subjects that underwent an ^18^F-flortaucipir PET scan within this sample, a partially overlapping sample of 107 amyloid-positive patients (47 females) aged between 55 and 94 years (mean age 75.4) and 139 amyloid-negative controls (78 females) aged between 56 and 94 years (mean age 73.6) who underwent at least one ^18^F-flortaucipir PET scan and one structural scan, all from ADNI, was used for tau-PET. The study subjects’ characteristics are summarized in *Table 1*. MRI and PET acquisition procedures for ADNI can be found in the online manual (http://adni.loni.usc.edu/methods/documents/mri-protocols). Briefly, structural MRI was recorded using a magnetization-prepared rapid gradient echo sequence (MP-RAGE) or an inversion recovery-fast spoiled gradient recalled sequence. Voxel size across protocols was 1.2 × 1.1 × 1.2 mm^3^ or 1 mm isotropic. For all participants, PET images were acquired as 5 min per frame, from 30 to 60 min post-injection for ^18^F-FDG, from 50 to 70 min post-injection for ^18^F-florbetapir and from 80 to 100 min post-injection for ^18^F-flortaucipir, respectively.

**Table 1:** ADNI samples characteristics

Subjects with structural MRI, FDG-PET and amyloid-PET	Controls Aβ− (*n* = 147)	Patients Aβ+ (*n* = 353)		
		Epicentres’ identification sample (*n* = 118)	Regression sample (*n* = 235)	Statistics
Age	73.98 ± 5.87	73.65 ± 7.20	73.41 ± 7.25	*H* = 0.151, df = 2, *P* = 0.927
Gender (male/female)	82/65	65/53	130/105	*χ* ^2^ = 0.014, df = 2, *P* = 0.993
Education	16.59 ± 2.38	15.85 ± 2.56	15.99 ± 2.77	*H* = 0,6.01, df = 2, *P* = 0.05
Dementia stage (EMCI/LMCI/AD)	NA	45/34/39	87/70/78	*χ* ^2^ = 0.051, df = 2, *P* = 0.974
MMSE	26.08 ± 8.87	23.76 ± 7.68	23.74 ± 8.06	*H* = 80.834, df = 2, *P* < 0.001
APOE-ɛ4 status (pos/neg)	26/121	71/46	175/60	*χ* ^2^ = 119.93, df = 2, *P* < 0.001

Subjects with tau-PET	Controls Aβ− (*n* = 139)	Patients Aβ+ (*n* = 107)		
		Epicentres’ identification sample (*n* = 32)	Regression sample (*n* = 75)	Statistics
Age	73.59 ± 7.75	74.61 ± 9.34	75.72 ± 7.36	*H* = 4.591, df = 2, *P* = 0.101
Gender (male/female)	61/78	17/15	43/32	*χ* ^2^ = 3.753, df = 2, *P* = 0.153
Education	16.29 ± 2.51	15.75 ± 2.50	15.64 ± 2.55	*H* = 4.61, df = 2, *P* = 0.1
Dementia stage (EMCI/LMCI/AD)	NA	12/15/5	32/32/11	*χ* ^2^ = 0.25, df = 2, *P* = 0.883
MMSE	29.13 ± 1.22	24.25 ± 4.84	24.88 ± 4.17	*H* = 96.022, df = 2, *P* < 0.001
APOE-ɛ4 status (pos/neg)	36/99	20/11	46/26	*χ* ^2^ = 33.39, df = 2, *P* < 0.001

Aβ−, amyloid-negative; Aβ+, amyloid-positive; df, degrees of freedom; EMCI, early mild cognitive impairment; LMCI, late mild cognitive impairment; NA, not applicable; MMSE, mini-mental state examination; pos, positive; neg, negative. Age, education and MMSE are represented by means and standard deviations and the statistics of the Kruskal–Wallis *H* test. Dementia stage, gender and APOE-ɛ4 status are represented by the number of subjects and the statistics of Pearson’s χ^2^ test. Some subjects have missing APOE-ɛ4 status.

#### Middle-aged healthy adults

Brain networks in healthy individuals have routinely been used to infer connectivity pathways shaping Alzheimer’s disease vulnerability patterns. Here, two datasets of right-handed healthy adults aged between 40 and 60 years were used to characterize the connectome and the anatomy of the healthy brain in middle adulthood.

##### Cam-CAN dataset

Diffusion-weighted images together with T_1_-weighted and T_2_-weighted images from the Cambridge Centre for Aging Neuroscience (Cam-CAN) repository^22-24^ were used to infer the structural connectome. Only right-handed middle-aged participants were included in the present study, resulting in 105 subjects (mean age 50.3, 53 females). The Cam-CAN study was conducted in compliance with the Helsinki Declaration and has been approved by the local ethics committee, Cambridgeshire 2 Research Ethics Committee (reference: 10/H0308/50). T_1_-weighted images were obtained using a 3D MP-RAGE, and T_2_-weighted images were obtained using a spatially selective single-slab 3D turbo-spin-echo (3D SPACE) sequence; both structural MRI sequences had 1 mm isotropic resolution. Diffusion-weighted images were acquired with a twice-refocused spin echo sequence using 2 mm isotropic spatial resolution and 30 gradient directions for each of two *b*-values: 1000 and 2000 s/mm^2^, plus three images acquired with a *b*-value of zero.

##### IMAP dataset

T_1_-weighted and functional MRI images from 48 right-handed native French-speaking healthy participants (mean age 49.3, 27 females) from the ‘Imagerie Multimodale de la Maladie d’Alzheimer à un stade Précoce’ (IMAP) study (Cyceron, France) were used to extract the functional connectivity and the inter-regional cortical distance networks. The IMAP study was approved by a regional ethics committee (Comité de Protection des Personnes Nord-Ouest III) and is registered with http://clinicaltrials.gov (number NCT01638949). All participants gave written informed consent to the study prior to the investigation. Some of these images were included in previous publications from our laboratory.^13,20,25-28^ No participant had a history of alcoholism, drug abuse, head trauma or psychiatric disorder. All included participants were amyloid-negative based on their global standardized uptake value ratio to the previously established threshold (1.11).^20,21^ Structural MRI was acquired using a 3D fast-field echo (3D-T1-FFE sagittal) sequence with the following parameters: repetition time = 20 ms, echo time = 4.6 ms, flip angle = 10°, 180 slices with no gap, slice thickness = 1 mm, field of view = 256 × 256 mm^2^ and in-plane resolution = 1 × 1 mm^2^. The resting-state functional MRI sequence was an axial interleaved 2D-T2*-sensitivity-encoded single-shot echo planar imaging (SENSE-EPI) sequence with SENSE = 2, repetition time = 2382 ms, echo time = 30 ms, flip angle = 80°, 42 slices of thickness 2.8 mm and with no gap, field of view = 224 *×* 224 mm^2^, in-plane resolution = 2.8 *×* 2.8 mm^2^, 280 volumes and acquisition time = 11.5 min. Subjects were instructed to keep their eyes closed during the scan.

### Data preprocessing

#### ADNI structural MRI and PET imaging preprocessing

Structural MRI images were segmented, normalized to the standard Montreal Neurological Institute (MNI) space, modulated using the Statistical Parametric Mapping^29^ (SPM) 12 software segmentation procedure and smoothed with an 8 mm full width at half maximum Gaussian filter. All downloaded PET images were averages of the dynamic volumes preprocessed by the ADNI PET Core to have standard orientation, the same image volume size (160 × 160 × 96) and voxel size (1.5 × 1.5 × 1.5 mm^3^) and a spatial resolution of 8 mm full width at half maximum. SPM and PYTHON were used for further processing of the downloaded PET images. All PET images were co-registered to their corresponding structural MRI images and normalized to the MNI template. The resulting ^18^F-FDG, ^18^F-florbetapir and ^18^F-flortaucipir PET images were intensity normalized using the cerebellar grey matter as a reference region. For the tau-PET sample, choroid plexus segmentation was performed using the ‘recon-all’ procedure of FreeSurfer^30^ 6.0 on structural images. This segmentation was then improved using Gaussian Mixture Models,^31^ and the delineated choroid plexus for each subject was finally transformed to MNI space.

#### Cam-CAN diffusion MRI preprocessing

Diffusion-weighted images were first preprocessed with FSL^32^ 5.0.10 leveraged through Nipype^33^ 0.13.0-gb770e8d32.dev to correct artefacts induced by head motion and eddy currents.^34^ Bias field correction of the diffusion-weighted and T_1_-weighted images was performed using ANTs N4^35^ 2.3.2. T_2_-weighted images were co-registered to their corresponding T_1_-weighted images using FLIRT^36,37^ in FSL with the boundary-based registration cost function. Both T_1_-weighted and T_2_-weighted images were used to estimate the deformation parameters from the subject’s space to the MNI template, then the T_1_-weighted image was co-registered to the average b0 image. The preprocessed images were exported to MRtrix 3.0 image format^38^ for fibre tracking. Response functions were estimated for the 2000 s/mm^2^ shell using a reimplementation of the iterative approach proposed in Tournier *et al*.^39^ Constrained spherical deconvolution^40^ was used to estimate a white matter fibre orientation distribution function. The co-registered T_1_-weighted image was tissue segmented, and 10 million streamlines seeded from the grey matter–white matter interface were generated using anatomically constrained tractography.^41^ A subset of 1 million streamlines was selected using the spherical-deconvolution informed filtering of tractograms approach,^42^ allowing the use of streamline count as a valid biological marker of connection density.

#### IMAP functional MRI preprocessing

Data were preprocessed as described in La Joie *et al*.^26^ with slice timing correction, realignment to the first volume and partial normalization within the native space to correct for distortion effects.^43^ Functional volumes were then co-registered to their corresponding structural image, normalized to the MNI space and smoothed with an isotropic 4 mm full width at half maximum Gaussian kernel.

#### IMAP structural MRI preprocessing

FreeSurfer was used for subsequent structural MRI preprocessing. Cortical surface reconstruction and subcortical segmentation were performed using the full version of ‘recon-all’. Because of the particular role of the allocortex in Alzheimer’s disease, we also reconstructed the hippocampus surface by tessellating its volume using the ‘mri_mc’ command and smoothing the obtained tessellation using the ‘mris_smooth’ command.

### Quantification of pathologies

#### Determination of individual patients’ biomarkers patterns

To assess atrophy, hypometabolism and amyloid-β and tau loads, W-score maps were computed for each patient using the healthy elder group as a reference as described in La Joie *et al*.^20^ W-scores are analogous to Z-scores, but they are adjusted for specific covariate(s).^44,45^ Covariates in the present study included age and education for all biomarkers and additionally total intracranial volume for atrophy (Supplementary Fig. 1A) and non-specific choroid plexus binding for tau.

#### Identification of brain maximal pathology sites

ADNI patients were randomly assigned to one of two samples while keeping the same proportions of early MCI, late MCI and Alzheimer’s disease patients: a one-third ‘maximal pathology sites selection sample’ and a two-third ‘regression sample’ dedicated to the cross-validated regression and relative importance analyses (Supplementary Fig. 1). The maximal pathology site selection sample was used to compute a representative grey matter mask and to identify bilateral brain regions showing maximal abnormality across the four modalities. The grey matter mask was obtained from normalized grey matter segments of the one-third patients’ sample and all the control subjects. A one-sample Student’s *t*-test was then performed on the W-maps of the considered patients to estimate the statistical significance of the biomarkers at the group level. T-maps were thresholded at Bonferroni-corrected *P* < 0.05 and cluster size *k* ≥ 50 voxels (168.75 mm^3^). The function ‘get_clusters_table’ implemented in Nilearn^46^ 0.8.0 was used to create global and local maxima. These maxima were subsequently used to define maximal pathology sites used as proxies for biomarkers’ epicentres: the locations of the first global and up to three local maxima of the thresholded T-maps, as well as their contralateral regions, were first selected (Supplementary Table 1), then the Brainnetome atlas^47^ was used to delineate the maximal pathology regions while discarding the basal nuclei (Supplementary Fig. 2).

#### Determination of group-level regional biomarkers

Repeated random sub-sampling was used to generate 1000 splits of the regression sample into independent training and test sets of the same size. Atrophy, hypometabolism and amyloid-β and tau loads T-maps for each training and test set were computed as explained previously. Regional biomarkers T-scores were computed as the average of positive voxel-wise *T*-values within each Brainnetome parcel after the intersection with the representative grey matter mask (Supplementary Fig. 1D).

### Healthy middle-aged brain characteristics

#### Structural connectome

For each subject from the Cam-CAN cohort, the atlas was transformed to diffusion-weighted space with SPM12, and the reconstructed streamlines were assigned to the parcels by a radial search outwards from the streamline termination point, out to a maximum radius of 2 mm as in Smith *et al*.^48^ To extract the structural connectome, a binary backbone network was estimated by testing each possible connection for its significance using the non-parametric sign test proposed in Gong *et al*.^49^ False discovery rate correction was applied to correct for multiple comparisons. The weighted network was then constructed by assigning to each edge a weight computed as the average across all subjects of the total number of tracked fibres between two regions divided by the mean volume of the two regions. This effectively normalizes the connection strength per unit volume and minimizes the region size-related bias.^50^

#### Functional connectome

Functional connectivity analysis was performed using Nilearn. Because region size strongly impacts functional connectivity strength,^51^ it is important to make region size homogeneous. The largest regions of the Brainnetome atlas (volume more than 2000 mm^3^) were, therefore, restricted to their most functionally relevant voxels. To this end, a group-independent components analysis^52,53^ was performed to extract resting-state networks from the IMAP individuals. Components were inspected to remove those related to physiological, movement or imaging artefacts. For each atlas region intersecting resting-state network components, components were incrementally thresholded until the overlap reached down as close as possible to 2000 mm^3^. The atlas regions that did not intersect any resting-state component were eroded to best match the volume of functional regions. This resulted in a selection of parcels of approximately equal volume within the Brainnetome regions (mean 1938, SD 397, in mm^3^). Voxel-wise signal was then averaged within each parcel intersected by the individual grey matter mask. To account for signals of non-neural origin, five principal components were extracted from voxel-wise signals within eroded white matter and cerebrospinal fluid individual masks,^54^ 24 motion confounds^55^ and a linear trend were regressed out from the raw time series for each parcel. Individual resting-state functional MRI correlation matrices were estimated using the shrunk estimator of Ledoit and Wolf^56^ as recommended by Brier *et al*.^57^ and Varoquaux *et al*.^58^ A one-tailed one-sample Student’s *t*-test was performed on the Fisher’s *z*-transformed correlation coefficients, and the resulting false discovery rate-corrected *P*-values were used to zero out non-significant correlations in the group average correlation matrix.

#### Inter-regional cortical distance network

To account for the confounding effect of cortical distance,^59^ we used structural MRI images from the IMAP study to measure distances between brain regions along the cerebral sulci and gyri. The cortical distance was measured by the exact geodesic distance along the pial (outer) cortical surface, calculated as the cumulative length of the shortest paths between two vertices. For each subject, the FreeSurfer version of the Brainnetome atlas was mapped to its native space to assign a regional label to each cortical surface vertex. The outer surface of the hippocampus was extracted and subdivided into rostral and caudal parts that matched the volumetric Brainnetome rostral and caudal hippocampus in a semi-automatic fashion using an in-house code that depended on PyVista^60^ 0.27.4 for mesh analysis. The geodesic distance was estimated between every pair of vertices within the cortical Brainnetome regions using SurfDist^61^ 0.15.0, ensuring that the considered shortest paths do not pass through non-cortical areas such as the ‘medial wall’. Exact geodesic distances between rostral and caudal hippocampal vertices were computed using tvb-gdist^62-65^ 2.1.0. Approximate geodesic distances between vertices lying on cortical Brainnetome regions and those lying on the hippocampal regions were computed by pairing the nearest vertices in the pial cortical surface and the hippocampus. For each pair of source and target regions, the minimal geodesic distance from every vertex on the surface mesh of the source region to the target region was computed, and distances were averaged to produce the distance from the source region to the target region. Since the geodesic distance is impacted by the size of the source region, only a fixed number of shortest geodesic paths was considered for large source areas. This procedure was repeated across all pairs of source and target regions and yielded two directed distance matrices that were then summed up to produce a symmetric undirected distance matrix. The group-level cortical distance matrix was obtained by averaging individual matrices across all subjects.

### Computation of pathologies predictors and confounding variables

Three graph-theoretical metrics reflecting possible spreading mechanisms were extracted from each network. The first graph metric consisted of nodal strength, defined as the sum of the weights of edges connecting a given region. Nodal strength is the simplest measure of centrality or ‘hubness’, and we used it here as a proxy for nodal stress, that is, the relatively increased intrinsic vulnerability of highly connected network elements.^66^ Nodal strength was computed for the structural and functional networks as the sum of the connection weights of each region.

The second graph measure we extracted was the diffusion mode, the long-term accumulation pattern of an agent diffusing between brain regions with rates proportional to connection weights.^7^ The diffusion mode of each connectome was estimated as the unsigned eigenvector associated with the lowest non-zero eigenvalue of its graph Laplacian, following the procedure described in Raj *et al*.^7^

The third graph metric depicted biomarker-specific topological proximity of a given region to the maximal pathology sites (epicentres’ proxies). This proximity measure captures how efficiently regions can be accessed from purported disease-onset sites. For each biomarker, the structural and functional connectomes were used to compute minimum path lengths to the epicentres after transforming connectivity values into distances by inversion. Topological proximity to the epicentres was computed as the inverse of the average shortest path length across all the epicentres.

Similar features were extracted from the inter-regional cortical distance network and formed confounding variables: nodal strength and diffusion mode were obtained after inverting non-zero cortical distances, and average shortest path lengths to the maximal pathology regions along the cortex were computed for each biomarker. Note that the diffusion mode for the cortical distance network was computed as the sum of the two modes characterizing the diffusion through each hemisphere. Predictors and confounding variables resulted for each biomarker in a total of nine regressors: six biomarker-independent regressors reflecting connectional and anatomical characteristics of each region and three biomarker-specific regressors summarizing the topological and cortical proximity to the maximal pathology sites (Supplementary Fig. 1C). Basal nuclei and parcels smaller than 100 voxels (337.5 mm^3^), as well as their contralateral regions, were discarded, resulting in a total of 228 regions.

### Statistical analyses

Results were considered significant at Bonferroni-corrected *P* < 0.05 unless specified otherwise.

#### Cross-validated regression and relative importance analyses

We developed a new framework that allows estimating the proportion of explained variance attributed to each independent variable in a cross-validated regression model. The predefined regressors were rescaled to have a mean of zero and a standard deviation of one and were used to predict regional biomarkers. For each imaging biomarker, the severity of the multicollinearity of the model was assessed through variance inflation factors (Supplementary Fig. 3 and Table 2), and as a remedy, regularized Ridge regression^67^ was performed. The statistical robustness and predictive power of the introduced model were tested through cross-validation using the two-third regression subsample. For each training/test split, the following steps were taken: (i) the regional T-scores for the training and test sets were rescaled to 0 mean and 1 SD; (ii) regularized Ridge regression was performed with the centered and standardized T-scores of the training set as the dependent variable, and the nine centered and standardized regressors as independent variables. The penalty was kept to the default value of the scikit-learn package (*λ* = 1) and (iii) the explained variance *R*^2^ of the so-derived model was computed on the test set based on the relative feature weight. All these steps were performed using scikit-learn^68^ 0.22.

The explained variance *R*^2^ for each pair of training/test sets was then partitioned between all the regressors by extending the Lindeman, Merenda and Gold (LMG) metric^69^ introduced for linear regression to regularized Ridge regression in a cross-validation setting. LMG quantifies the proportionate contribution of each regressor to the variance explained by the model by comparing models containing all possible subsets of independent variables.^70^ Following Grömping, for a given permutation *r* of the regressors’ indices {1, …, *m*} (*m* = 9 in our case), the improvement in explained variance when adding the regressor *X*_1_ to a model that already contains the regressors appearing before *X*_1_ in the order *r* was defined as


$$\mathrm{svar}(\left\{1\}|S(r\right))=\mathrm{var}(Y|X_{j},j\in S(r))-\mathrm{var}(Y|X_{j},j\in S(r)\cup \{1\}),$$

where *S*(*r*) denotes the set of indices preceding one in *r*. The LMG contribution of *X*_1_ to the variance explained by the complete model was set to the average over allocations to *X*_1_ from all possible ordering of regressors.


$$\mathrm{LMG}(X_{1})=\frac{1}{m!}\underset{r\;\mathrm{permutation}}{\sum }\;\mathrm{svar}(\left\{1\}|S(r\right)).$$

However, unlike Grömping, we fit the models on the training sets using regularized regression, and we evaluated the explained variances on the corresponding test sets so that the LMG contributions sum up to the cross-validation *R*^2^. For each regressor, the average of the LMG contributions over the 1000 test sets was used as a pointwise estimate of the proportion of variance it explains.

#### Bootstrap confidence intervals

Ninety-five per cent confidence intervals for the LMG contributions were obtained using the strategy of Timoshenko to efficiently and accurately bootstrap cross-validation performance estimates.^71^ Specifically, 10 000 bootstrap replicates of the predicted regional biomarkers were generated by sampling 228 (the total number of regions) pathology predictions with replacement. Predictions on the same region for different training/test splits were all included in a bootstrap sample or none at all. Predictions that were not selected in a bootstrap sample were used to estimate the average LMG contributions over the 1000 test sets. For each regressor, the 95% CI lower and upper bounds of its LMG contribution were set to the 2500th lowest and 9750th largest values of its 10 000 bootstrap estimates, respectively.^72^ Contributions with a non-negative 95% CI lower bound were considered significant.

#### Comparison of relative contributions between APOE-ɛ4-positive and negative patients

One hundred and six APOE-ɛ4-negative ADNI patients (37 for the tau-PET sample) were matched with the same number of APOE-ɛ4-positive patients on age, sex, education and clinical status. A two-tailed Welch’s *t*-test was performed to test for significant differences between mean biomarkers W-maps for carriers and non-carriers. The whole procedures of maximal pathology site selection, regression and estimation of LMG contributions were repeated with the APOE-ɛ4 status-specific samples. The T-maps obtained from the maximal pathology sites selection sample were thresholded using a Bonferroni-corrected significance cut-off and if no voxel survived this threshold, an uncorrected significance cut-off of *P* < 0.001 was used. The global and local maxima of the T-maps were computed, and when the number of maximal pathology sites was different between carriers and non-carriers, the second global maximum and related local maxima were used to reach the same number of epicentres’ proxies. Maximal pathology sites were paired between carriers and non-carriers based on the maximal statistic of their corresponding peaks. Levene’s test was used to test whether the variances of the regional biomarkers T-scores at paired maximal pathology sites were different between the APOE-ɛ4-positive and negative groups. A two-tailed Fisher–Pitman permutation test was performed between the LMG contributions of carriers and non-carriers; only contributions from structural and functional pathways with non-negative 95% CI lower bounds were compared.

### Visualizations

Surface brain visualizations were generated using SurfIce^73^ and the Connectome Workbench^74^ 1.4.2 software. Colours were chosen to be colour-blind-friendly using ColorBrower^75^ 2.0 and Color Cycle Picker.^76^

## Results

### Structural and functional topological descriptors showed partly distinct patterns

**Figure 1:**
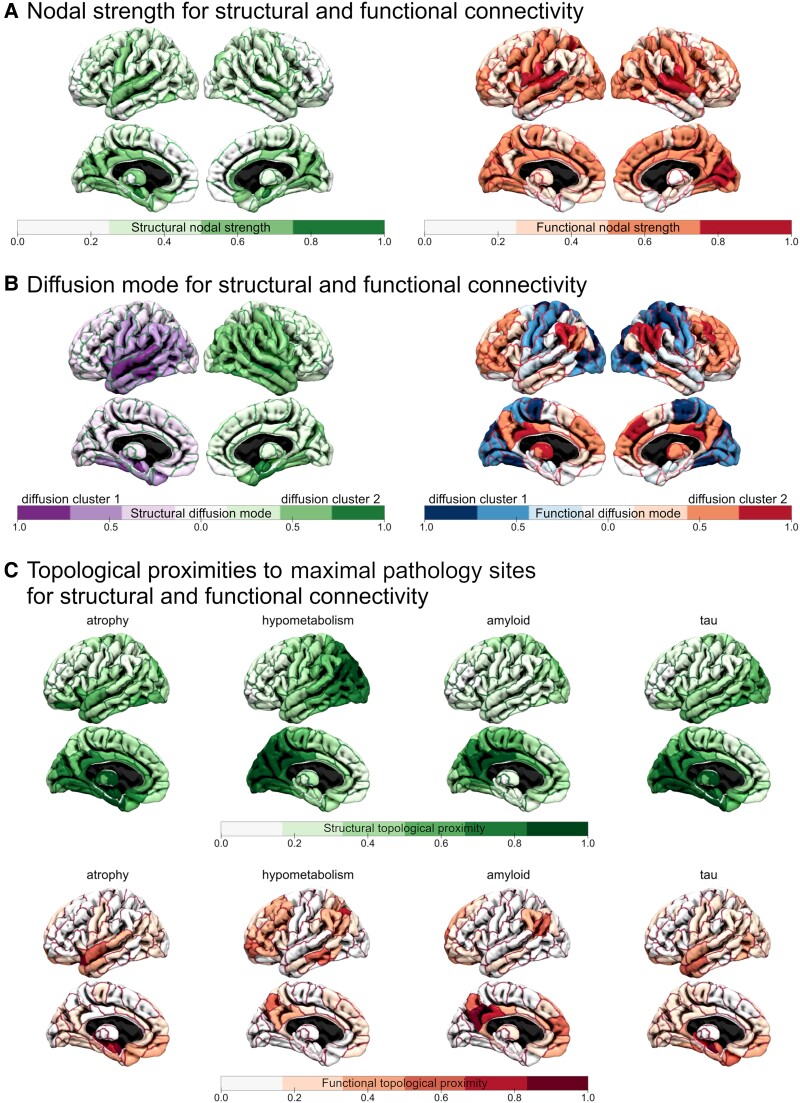
**Graph metrics extracted from structural and functional connectivity networks.** (**A**) Nodal strength for structural and functional connectivity. (**B**) Diffusion modes for structural and functional connectivity, with the associated diffusion clusters. (**C**) Topological proximity to the maximal pathology sites for structural and functional connectivity. Values have been linearly transformed to 0 minimum and 1 maximum to ease visual comparisons.

The correlation between the nodal strength of the structural and the functional connectomes was not significant (Pearson’s *r =* 0.1, *P* > 0.15). For both the structural and functional connectivity networks, many regions amongst the top 10% most strongly connected nodes lay in the occipital lobe, precuneus, insula and fusiform gyrus, but only three regions were common between structural and functional hubs: the ventromedial parieto-occipital cortex, ventral insula and lateroventral fusiform gyrus. Other regions ranking in the top 10% of most strongly connected nodes of the structural connectivity network were located in the orbital gyrus, anterior cingulate cortex, middle temporal gyrus, parahippocampal gyrus, hippocampus and amygdala (Fig. 1A, left). As for the functional connectome, the locations of the remaining hubs were the superior frontal gyrus, pre and postcentral gyri, superior parietal lobule, posterior cingulate cortex (PCC) and superior temporal gyrus (Fig. 1A, right). The above regions matched the consistent locations of structural^49^ and functional^77^ hubs across the adulthood lifespan.

The diffusion mode reflects the last local equilibrium stage before the diffusion process reaches stationarity, during which diffusion would occur more frequently within two minimally communicating communities than between them. We illustrated these two diffusion clusters and the magnitude of the diffusion mode in each brain region in Fig. 1B for the structural and functional connectomes. In agreement with studies of the structural network diffusion model, we found that the diffusion clusters of our structural connectome captured left–right gradients^78,79^ and that the diffusion mode showed strong involvement of medial and lateral temporal lobes^7^ (Fig. 1B, left). The diffusion clusters associated with the functional connectome were substantially different, roughly separating the default mode, frontoparietal and ventral attention networks from visual, somatomotor and dorsal attention networks, and basal ganglia regions from the hippocampus and amygdala (Fig. 1B, right). The corresponding diffusion mode mostly highlighted the lateral occipital lobe. The diffusion modes of the structural and functional connectivity networks were weakly anti-correlated (Pearson’s *r =* −0.38, *P* < 3 × 10^−8^).

### Structural and functional topological proximity to maximal pathology sites were weakly correlated

Multiple maximally affected regions and their contralateral counterparts were considered as proxies of epicentres for grey matter atrophy, hypometabolism and amyloid-β and tau deposition, respectively (Fig. 1C). These maximal pathology sites lay in the amygdala, hippocampus and temporal pole for atrophy; the angular gyrus and inferior temporal gyrus for hypometabolism; the PCC, angular gyrus and posterior gyrus rectus for amyloid-β and the hippocampus, fusiform gyrus and temporal pole for tau (Supplementary Table 1).

Topological proximity to the epicentres’ proxies through the structural and functional connectivity networks were moderately correlated for atrophy (Pearson’s *r* = 0.52, *P* < 3 × 10^−17^) and for amyloid-β (Pearson’s *r =* 0.43, *P* < 8 × 10^−12^) and tau loads (Pearson’s *r =* 0.54, *P* < 2 × 10^−18^); their correlation was not significant for hypometabolism (Pearson’s *r =* 0.09, *P* > 0.15) (Fig. 1C).

### Connectivity patterns shaped Alzheimer’s disease through concurrent mechanisms

**Figure 2:**
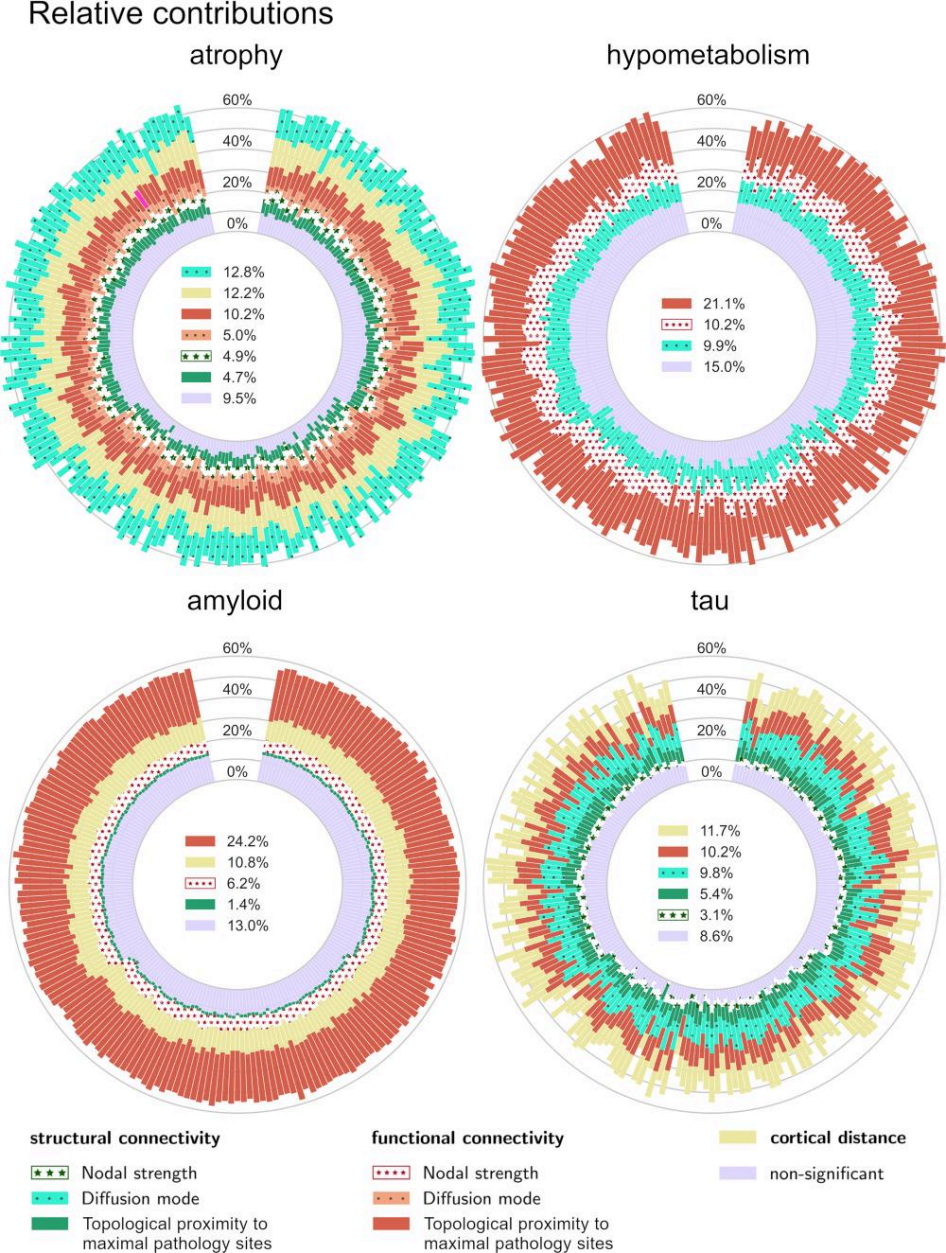
**Relative contributions.** Stacked bars represent the explained variance attributed to each predictor. Contributions with negative confidence intervals lower bound are grouped into the ‘non-significant’ bars. Significant contributions from the confounding variables (extracted from the cortical distance network) are grouped into the ‘cortical distance’ bars. Legend values are the average contributions across all cross-validation test sets. Fluctuations reflect variability across 200 random samples out of the 1000 cross-validation test samples. The contributors are ordered from interior to exterior according to their increasing magnitude of average contribution (except for the ‘non-significant’ bars placed at the bottom of the stacks). The maximal pathology sites are biomarker-specific and considered as proxies for epicentres.

Structural MRI images, ^18^F-FDG-PET and amyloid-PET images from 235 amyloid-positive patients and tau-PET images from 75 amyloid-positive patients, different from patients included in the maximal pathology sites selection samples, were randomly sub-sampled to generate 1000 independent training and test samples of regional patterns of Alzheimer’s disease biomarkers. The average overall out-of-sample variances explained by our models were 58.1% [95% confidence interval (95% CI): (42%, 70.1%)] for atrophy; 56.1% [95% CI (46.7%, 64.6%)] for hypometabolism; 55.4% [95% CI (44.3%, 64.9%)] for amyloid-β and 48.7% [95% CI (37%, 58.6%)] for tau. Nodal stress, diffusion and propagation from epicentres’ proxies through the structural and functional connectivity networks differently impacted the spatial distribution of each biomarker: significant contributors (with non-negative confidence intervals 95% CI lower bounds) differed across modalities and their average relative contributions ranged from 1.4% to 24.2% (Fig. 2).

The relative contribution explained by each predictor was robust across the different cross-validation training–test sets. More than 30% of the variance of hypometabolism and amyloid-β deposition patterns was explained by functional connectivity pathways, while structural connectivity pathways explained 22.4% of regional variability for atrophy and 18.4% for tau (Supplementary Table 2). Topological proximity to the maximal pathology sites through the functional network significantly contributed to predicting all biomarkers, the highest contribution being for amyloid-β deposition [24.2%, 95% CI (15.1%, 33.6%)]. It was also a major contributor to hypometabolism, with an explained variance of 21.1% [95% CI (11.3%, 30.2%)], followed by tau [10.2%, 95% CI (4.3%, 16%)] and atrophy [10.2%, 95% CI (1.9%, 16.4%)]. Diffusion along structural connections played a major role in shaping atrophy and tau deposition patterns, respectively explaining 12.8% [95% CI (7.6%, 18.4%)] and 9.8% [95% CI (3.7%, 15.6%)] of variance and ranking the first and second top contributing processes.

### APOE-ɛ4 carriage affected contributions consistently across all biomarkers

To investigate the effect of APOE-ɛ4 carriage on spreading, we matched 106 (37 for the tau-PET sample) APOE-ɛ4-negative patients to the same number of APOE-ɛ4-positive patients on age, sex, education and clinical status. APOE-ɛ4-positive patients showed higher hypometabolism and higher amyloid-β and tau loads, while atrophy was higher for APOE-ɛ4-negative patients (Welch’s *t*-test, *P* < 0.001 uncorrected), but differences did not survive multiple comparisons correction. Higher hypometabolism was restricted to two little regions in the superior frontal gyrus. Higher amyloid-β load was observed mainly in parts of the dorsal default mode network, while higher tau load was located in a few cortical and subcortical areas, including the anterior cingulate cortex, orbital gyrus, hippocampus, amygdala and nucleus accumbens. APOE-ɛ4-negative patients had widespread more atrophied regions in particular in the temporal and frontal lobes (Supplementary Fig. 4C).

The maximal pathology sites identification and the cross-validated procedure were repeated on each APOE-ɛ4 status-specific sample. Common maximal pathology regions lay in the caudal hippocampus for atrophy and PCC for amyloid-β deposition. Maximal pathology regions specific to APOE-ɛ4 carriers were located in the amygdala for atrophy; the inferior temporal, middle temporal and fusiform gyri for hypometabolism; the dorsolateral prefrontal cortex, dorsal PCC and superior occipital gyrus for amyloid-β deposition; and the amygdala, hippocampus and entorhinal cortex for tau deposition. Maximal pathology regions specific to non-carriers were located in the rostral hippocampus for atrophy; the hippocampus, occipital thalamus and lingual and angular gyri for hypometabolism; the medial parieto-occipital sulcus, ventral PCC and fusiform gyrus for amyloid-β deposition; and the lingual and angular gyri for tau deposition (Supplementary Table 3).

**Figure 3:**
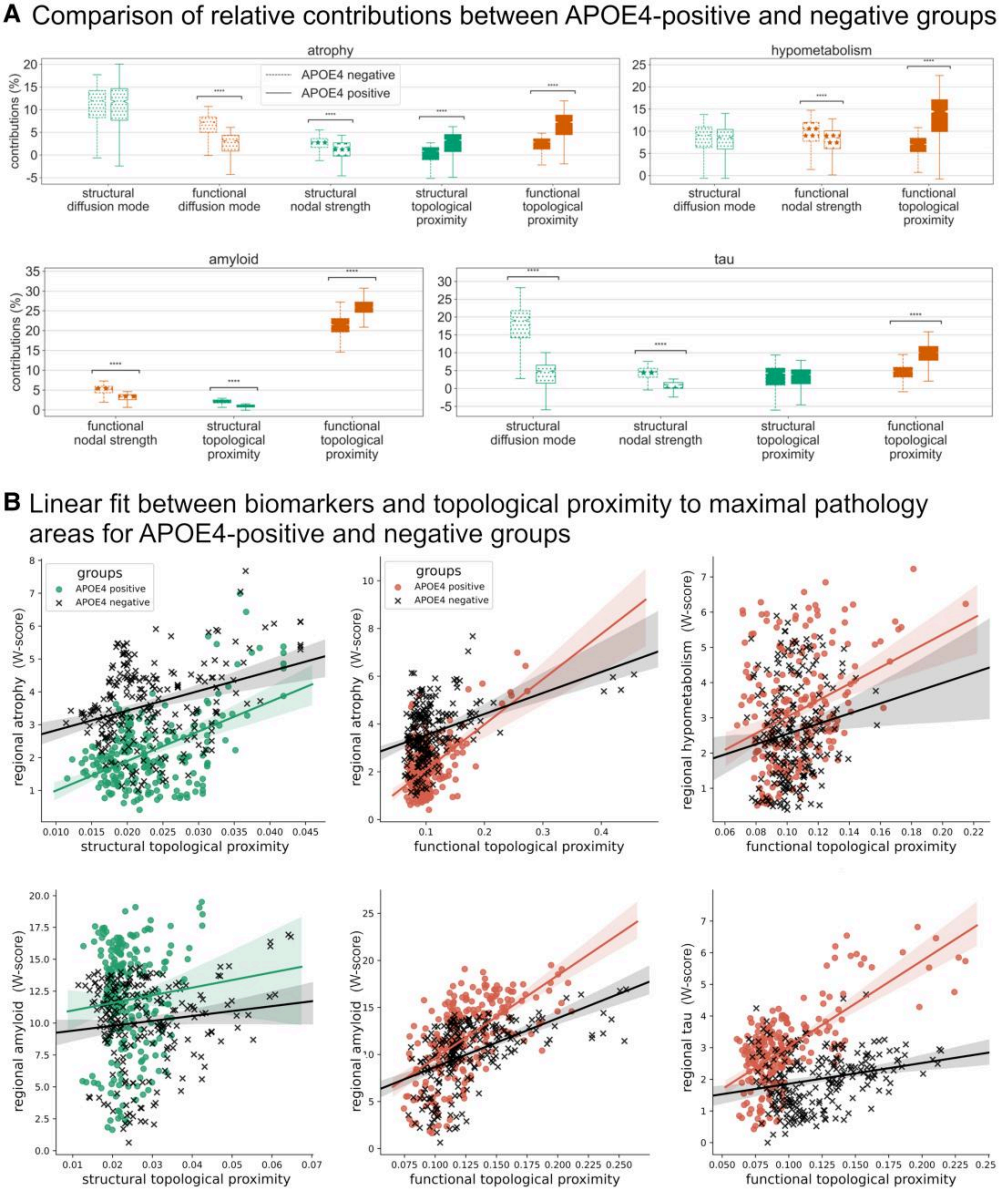
**Comparison of APOE-ɛ4-positive and negative groups.** (**A**) Comparison of predictors’ significant contributions. Participants (*n* = 212, except for the tau-PET sample where *n* = 74) were evenly distributed across the APOE-ɛ4 carrier and non-carrier groups. Groups were marched for age, sex, education and clinical status, and compared using Fisher–Pitman permutation test. *P*-values were corrected for multiple comparisons using the Bonferroni correction and are reported as *P*_corrected_ < 0.05 by multiplying the original *P*-value by the number of comparisons. Atrophy: stat = −0.46, *P*_corrected_ = 3.22; stat = −32.7, *P*_corrected_ < 0.000; stat = −15.1, *P*_corrected_ < 0.000; stat = 16.0, *P*_corrected_ < 0.000 and stat = 30.2, *P*_corrected_ < 0.000; for structural and diffusions modes, structural nodal strength, structural and functional topology proximities, respectively. Hypometabolism: stat = −1.45, *P*_corrected_ = 0.46; stat = −11.1, *P*_corrected_ < 0.000 and stat = 31.9, *P*_corrected_ < 0.000; for structural diffusion mode, functional node strength and functional topology proximity, respectively. Amyloid: stat = −38.4, *P*_corrected_ < 0.000; stat = −37.0, *P*_corrected_ < 0.000 and stat = 46.4, *P*_corrected_ < 0.000; for functional nodal strength, structural and functional topology proximities, respectively. Tau: stat = −52.3, *P*_corrected_ < 0.000; stat = −44.0, *P*_corrected_ < 0.000; stat = 0.12, *P*_corrected_ = 3.63 and stat = 40.9, *P*_corrected_ < 0.000; for structural diffusion mode, structural nodal strength, structural and functional topology proximities, respectively. (**B**) Linear fit between biomarkers and topological proximity to maximal pathology areas. Participants (*n* = 212 except for tau-PET were *n* = 74) were evenly distributed across the APOE-ɛ4 carrier and non-carrier groups. Biomarkers were estimated as W-score from all participants. Topological proximity was computed as the inverse of the average shortest path length across all the maximal pathology areas. APOE, apolipoprotein E.

For all imaging biomarkers, significant differences in the contributions of most of the spreading mechanisms were observed between APOE-ɛ4 carriers versus non-carriers (Fisher–Pitman permutation test, corrected *P* < 0.05, Fig. 3A). Except for the very weak contribution of the topological proximity to maximal pathology sites through the structural connectome to amyloid-β deposition (1.7% for the APOE-ɛ4-positive group, 3.7% for the APOE-ɛ4-negative group), the significant differences followed the same pattern: a decrease in the contributions of nodal strength and diffusion mode and an increase in the contributions of topological proximity to maximal pathology sites in the APOE-ɛ4-positive group.

Because APOE-ɛ4 non-carriers have more variability than carriers in Alzheimer’s disease subtypes,^80^ the observed lower contributions from topological proximity to epicentres’ proxies could be driven by a less homogeneous distribution of the imaging biomarkers across non-carriers, rendering the impact of epicentres less salient. To check this possibility, post hoc analyses were conducted on the whole APOE-ɛ4-negative sample and its matched APOE-ɛ4-positive sample. Using Levene’s test, we assessed whether the variances of the imaging biomarkers at paired maximal pathology sites were different between the two groups. No significant difference was found for all biomarkers (*P* > 0.1, corrected). Moreover, we observed that atrophy, hypometabolism and amyloid-β and tau loads increased more strongly for carriers than for non-carriers when getting close to the maximal pathology sites through the functional network (and through the structural network for atrophy, Fig. 3B). In summary, the increases in the contribution of topological proximity to epicentres in APOE-ɛ4 carriers did not result from a higher variability in the location of sites of maximal pathology in non-carriers but were likely driven by an excess of vulnerability in the topological vicinity of the epicentres.

## Discussion

Several mechanisms are likely involved in the spreading of Alzheimer’s disease pathology along connections, but little is known about their relative contributions and their differential contribution according to the pathology being studied. Here we provide a general framework for disentangling the relative contributions of specific connectional pathways to the topography of Alzheimer’s disease from concurrent mechanisms. Our study (i) provided a general cross-validated framework to estimate the proportion of variance explained by each predictor in multivariate regression models; (ii) combined structural and functional connections as distinct pathology spreading routes; (iii) controlled for the confounding effect of cortical distance; (iv) evaluated the relative contribution of nodal stress, diffusion and propagation from disease epicentres’ proxies along the considered pathways to the topography of atrophy, hypometabolism and amyloid-β and tau deposition in amyloid-positive patients and (v) tested for differences in contributions between APOE-ɛ4 carriers and non-carriers.

Previous connectome-based multivariate models^13,18,81^ reported the significant contributions of different spreading processes but used functional connectivity as a surrogate for structural connections. Studies investigating propagation along both structural and functional pathways have been inconclusive. For instance, Vogel *et al*.^9^ noted that their epidemic spreading model simulating tau-PET spread had a slightly better performance when using structural connections versus functional connections, while Franzmeier *et al*.^82^ found that restricting the functional connectome to structural connections did not improve the prediction of regional tau-PET uptake. Our study focused on leveraging the best-practice variance decomposition technique within an out-of-sample evaluation framework. Thereby, our approach enabled us to disentangle contributions from closely related pathways.

As regards structural versus functional pathways, we found two main results. First, we found that functional connectivity predictors explained substantial parts (31% of cumulated variance) of hypometabolism and amyloid-β deposition variances, while contributions from structural pathways were marginal (cumulated variance <10%). Second, an opposite albeit weaker trend was observed for atrophy and tau, with structural predictors, respectively, totalizing 20% and 17% of explained variance, and functional connectivity pathways cumulating 15% and 10% of it. Whereas structural brain networks reflect physical measures of neural wiring, functional connectivity reveals the pairwise similarity between brain regions’ dynamics. Although the neural mechanisms that give rise to blood-oxygen-level-dependent fluctuations are not yet clearly established, resting-state functional MRI signals have been consistently found to correlate to the electrophysiological signals arising from the integrated electrical activity in pre- and post-synaptic terminals of the brain. In turn, there is strong evidence *in vitro* and *in vivo* that the synaptic activity bidirectionally regulates the secretion of amyloid-β^83-85^ and modulates the depositions of amyloid-β.^86-89^ Increased activity has also been associated with enhanced tau pathology and tau propagation,^90,91^ and the rate of tau accumulation in distant regions from epicentres has been related to functional connectivity pathways.^82^ Accordingly, our results support the hypothesis that the trans-synaptic spread of amyloid-β and to a lesser extent that of tau may be related to synaptic activity itself. The disproportionately small contribution from structural connectivity to amyloid-β topographical variance in comparison with tau is in line with the observation that, unlike tau, amyloid-β deposits are rarely and only in small quantity found in the white matter in Alzheimer’s disease patients,^92^ and that amyloid-β propagation is associated with a dendrite-related genetic profile while tau propagation is associated with an axon-related genetic profile.^93^ The link between functional connectivity and hypometabolism is more straightforward since local metabolic activity and functional connectivity are physiologically coupled.^94^

As regards the spreading mechanisms, we found that the propagation from the maximal pathology sites was a major contributor to shaping the topography of all biomarkers, particularly that of amyloid-β and tau, for which it occurred through both structural and functional networks and totalized more than 26% of explained variance. Nodal stress also contributed to all markers but more modestly. Moreover, nodal strength had surprisingly a negative correlation with hypometabolism. Such an inverse relationship with functional nodal strength has been reported for atrophy^13^ but never for hypometabolism. The contribution of functional nodal strength to atrophy was not significant in our study, but their correlation was indeed negative. Since we did not apply a correction for partial volume effects, the negative association we observed may be due to its presence in atrophied areas. Nevertheless, the relationship between functional nodal stress and atrophy/hypometabolism warrants further investigation. Finally, our results confirmed the important role of diffusion on structural networks in shaping atrophy, hypometabolism and tau patterns. By contrast, diffusion did not contribute to amyloid-β deposition. Interestingly, network diffusion processes get trapped within tight-night communities and hardly reach low-interconnected nodes.^95^ This dynamic may explain why tau pathology, even though it may precede amyloid-β deposition^96-98^ remains mostly confined to medial temporal regions while amyloid-β distributes to all brain lobes.

When comparing APOE-ɛ4 carriers to non-carriers, we found significant decreases in the contributions from nodal strength and diffusion modes and significant increases in contributions from topological proximity to maximal pathology sites in carriers for all our imaging biomarkers, except for a significant decrease in the very weak contribution of the topological proximity through the structural network to amyloid-β deposition. Notably, these directions of effects are consistent with compromised brain connectivity in APOE-ɛ4 carriers, with the intrinsic vulnerability of network elements systematically explaining less variance, and pathology being more confined to the topological vicinity of the maximal pathology sites. The ɛ4 allele of the APOE gene affects the integrity of white matter microstructure^99,100^ through suboptimal myelin repair/remyelination,^101^ resulting in disrupted brain connectivity. Middle-aged and older asymptomatic APOE-ɛ4 carriers show therefore age-related loss of local structural connectivity^102^ and weakened default mode network functional connectivity.^103-106^ Our findings suggest that the differential vulnerability of brain regions in APOE-ɛ4 carriers compared with non-carriers mirrors the alteration of their communication pathways. Distinct spread between APOE-ɛ4 carriers and non-carriers might, therefore, be related to their specific connectomic profile, but this hypothesis requires further investigation on the exact impact of APOE-ɛ4 on the spreading mechanism.

The observations reported in this study are purely phenomenological and do not intend to prove biological mechanisms. We interpreted our results under the hypothesis of trans-neuronal and trans-synaptic spread of amyloid-β and tau, but other mechanisms can account for our findings. Correlated gene expression is found among connected regions of structural^107,108^ and functional^109,110^ connectivity networks. The contributions of connectional pathways to explaining the patterns of Alzheimer’s disease markers may thus, in part, result from shared genetic vulnerability to developing Alzheimer’s disease pathology.^93,111^ Moreover, the results were assessed using parametric linear modelling, and the interpretation was based on a weighted beta coefficient designed for prediction analysis. Non-linear machine learning algorithms will be here of relevance for further investigations.^70^ We defined the maximal pathology sites based on statistical thresholds, but further investigations on the optimal number of sites used as epicentre proxies will be necessary in order to increase reproducibility.

Our methodological framework is readily applicable to other settings for measuring relative contributions from related predictors. Because it relies on cross-validation and bootstrapping on a large database, we expect our findings to be generalizable to other datasets. Replication on independent cohorts would be the next step to confirm replicability.

## Supplementary Material

fcae459_Supplementary_Data

## Data Availability

ADNI and Cam-CAN datasets are available at https://adni.loni.usc.edu and https://cam-can.mrc-cbu.cam.ac.uk/dataset, respectively. The authors may share de-identified data and codes upon request to the corresponding author G.C. All applicants will be asked to sign a data access agreement.
