# Supplementary material for: Contributions of connectional pathways to shaping Alzheimer’s disease pathologies
Source: Brain Commun. 2025 Jan 6;7(1):fcae459. doi: 10.1093/braincomms/fcae459 (PMC11702304; doi:10.1093/braincomms/fcae459)
Supplement: fcae459_Supplementary_Data [file fcae459_supplementary_data.docx]

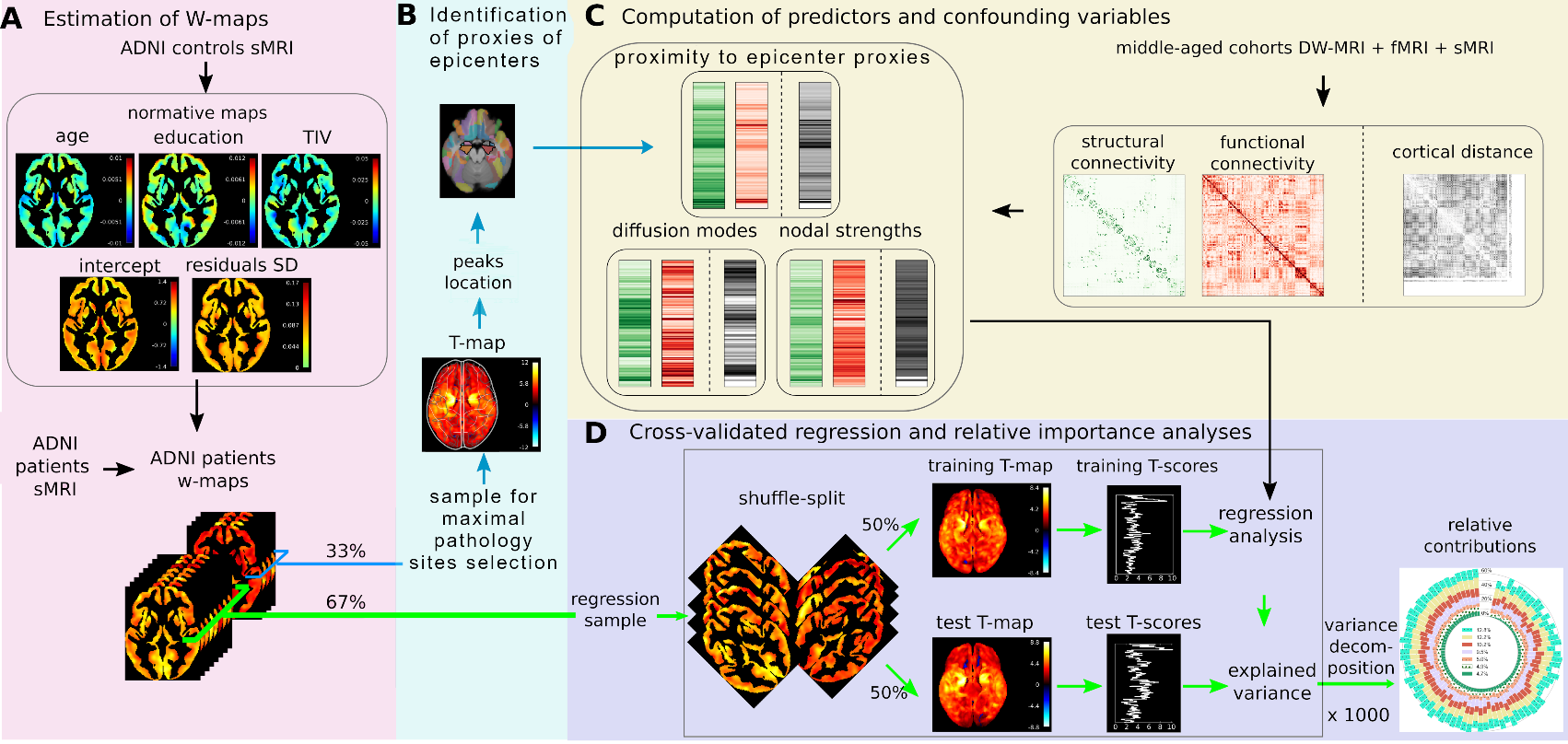
**Supplementary Figure 1. Study design**

(**A**) Estimation of atrophy W-maps. (**B**) Identification of atrophy maximal pathology sites used as proxies of epicenters. (**C**) Estimation of atrophy predictors and confounding variables. (**D**) Cross-validation with repeated shuffle split, model fitting for each train set, and decomposition of the explained variance for each test set. ADNI = Alzheimer’s disease neuroimaging initiative; DW-MRI = diffusion-weighted MRI; fMRI = functional MRI; sMRI = structural MRI; SD = standard deviation; TIV = total intracranial volume

**Supplementary Table 1. Identification of maximal pathology sites: coordinates of the first global and up to three local maxima and peak T-statistics of the thresholded T-maps.**

| **Pathology** | **Global maximum**  **location** | **Local maximum**  **location** | **Side** | **MNI coordinates** | | | **Brainnetome region** | **Peak T** |
| --- | --- | --- | --- | --- | --- | --- | --- | --- |
|  |  |  |  | **x** | **y** | **z** |  |  |
| Atrophy | Amyg |  | R | 21 | -6 | -14 | mAmyg, medial amygdala | 9.9 |
|  |  | cHipp | R | 16 | -36 | 3 | cHipp, caudal hippocampus | 7.3 |
|  |  | TP | R | 45 | 6 | -18 | A38l, lateral area 38 | 6.8 |
|  |  |  |  |  |  |  |  |  |
| Hypometabolism | AG |  | L | -42 | -63 | 42 | A39rd, rostrodorsal area 39 (Hip3) | 10.1 |
|  |  | iTG | L | -58 | -31 | -21 | A20cl, caudolateral of area 20 | 9.9 |
|  |  | AG | L | -46 | -58 | 32 | A39rv, rostroventral area 39 (PGa) | 9.5 |
|  |  | iTG | L | -56 | -50 | -15 | A20cl, caudolateral of area 20 | 9.5 |
|  |  |  |  |  |  |  |  |  |
| Amyloid | PCC |  | R | 3 | -54 | 30 | A31, area 31 (Lc1) | 25.6 |
|  |  | AG | L | -52 | -58 | 14 | A39rv, rostroventral area 39 (PGa) | 23.2 |
|  |  | pGR | R | 3 | 38 | -14 | A14m, medial area 14 | 23 |
|  |  | dPCC | L | 0 | -26 | 39 | A23d, dorsal area 23 | 22.7 |
|  |  |  |  |  |  |  |  |  |
| Tau | rHipp |  | L | -21 | -4 | -27 | rHipp, rostral hippocampus | 8.5 |
|  |  | cHipp | L | -28 | -33 | -12 | cHipp, caudal hippocampus | 6.5 |
|  |  | TP | L | -28 | 6 | -40 | A38m, medial area 38 | 6.2 |
|  |  | FuG | L | -42 | -45 | -12 | A37lv, lateroventral area 37 | 6 |

MNI coordinates are rounded to the nearest integer. R = right; L = left; AG = angular gyrus; Amyg = amygdala; cHipp = caudal hippocampus; rHipp = rostral hippocampus; dPCC = dorsal posterior cingulate cortex; FuG = fusiform gyrus; iTJ = inferior temporal gyrus; MNI = Montreal national institute; PCC = posterior cingulate cortex; pGR = posterior gyrus rectus; TP = temporal pole.

**Supplementary Figure 2. Maps of the maximal pathology sites for the 4 biomarkers.**


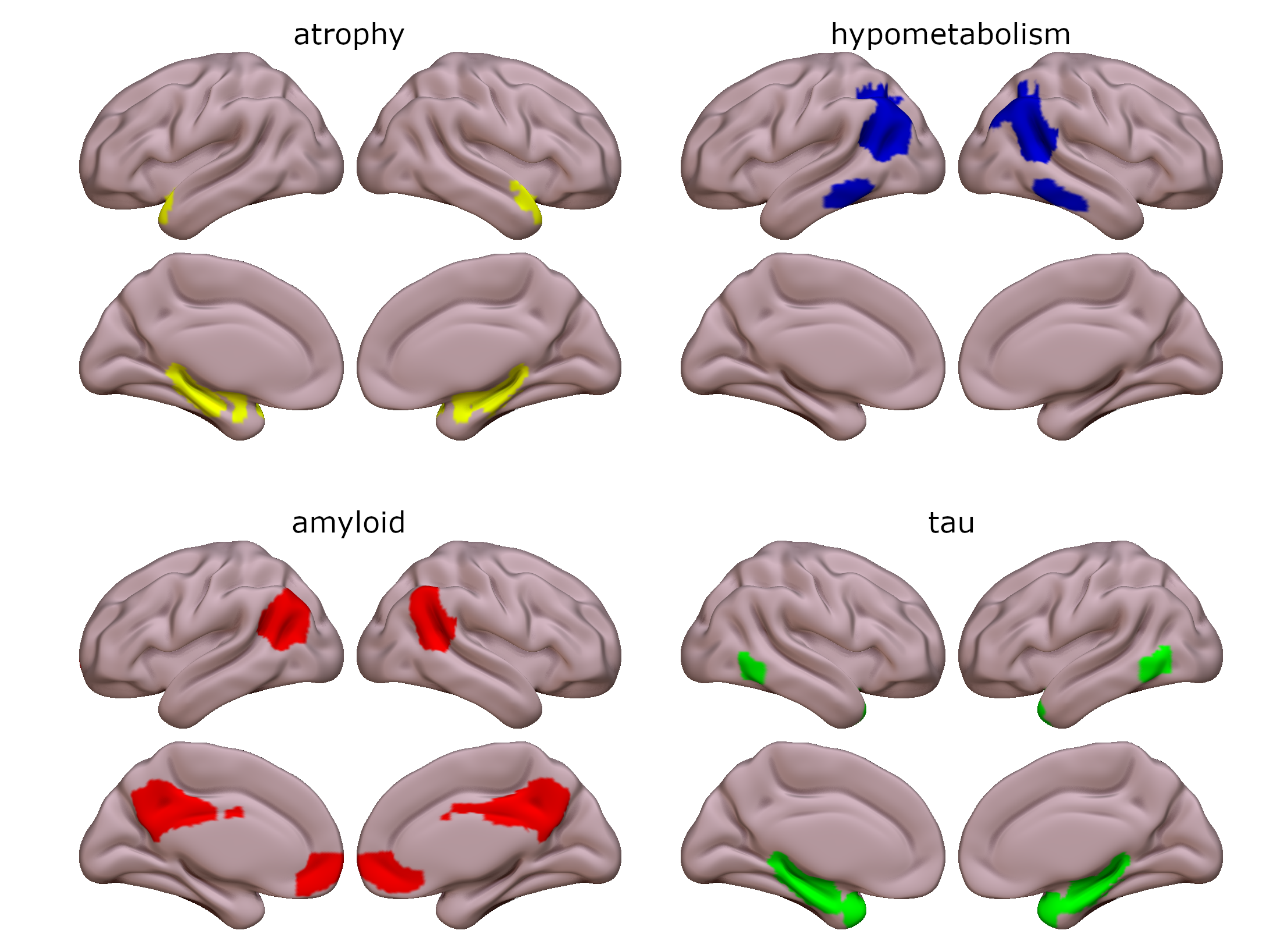


Maximal pathology sites were bilaterally delineated using the Brainnetome Atlas, based on the significant clusters provided in Supplementary Table 1. Peak of atrophy included the lateral part of the superior temporal gyrus (label 77/78), the caudal hippocampus (label 217/218), and the medial amygdala (label 211/212). Peak of hypometabolism included the caudolateral part of the inferior temporal gyrus (label 99/100), the rostroventral and rostrodorsals part of the inferior parietal lobule (label 143/144 and 137/138, respectively). Peak of amyloid deposition included the medial part of the orbitofrontal gyrus (label 41/42), the dorsal part of the cingulate gyrus (label 175/176), the area 31 of the precuneus (label 153/154), and the rostroventral part of the inferior parietal lobule (label 143/144). Peak of tau deposition the ventrolateral part of the inferior temporal gyrus (label 97/98), the medial part of the superior temporal gyrus (label 69/70), and the rostral and caudal hippocampus (label 215/216 and 217/218, respectively).

**Supplementary Figure 3. Correlation matrices between connectome features for the 4 biomarkers.**


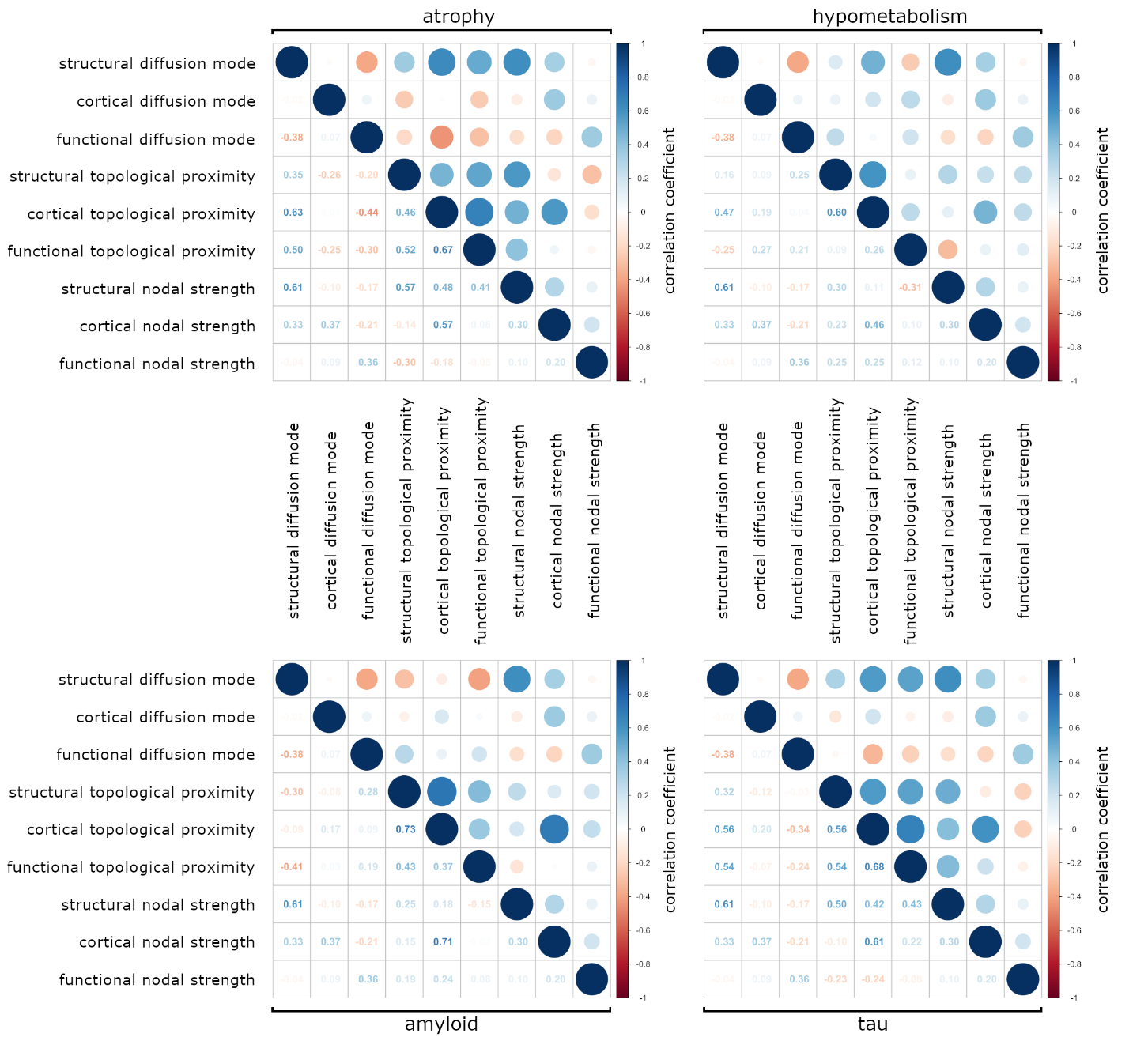


Correlation coefficients are Pearson’s coefficients, expressed both as numeric values (south-west part of the matrix) and graphic circle representations (north-east part of the matrix). Proximity refers to proximity to maximal pathology sites used as proxies of epicenters. N = 228 brain areas.

**Supplementary Table 2. Relative contributions.**

| **Pathology** | **Regressors** | | **VIF** | **Pearson's r** | **LMG** | | |
| --- | --- | --- | --- | --- | --- | --- | --- |
|  |  |  |  |  | **Mean** | **SD** | **95% CI** |
| Atrophy |  | |  |  |  |  |  |
|  | Structural connectivity | |  |  |  |  |  |
|  |  | Nodal strength | 2.82 | 0.41 | 4.94% | 1.08% | 0.70%, 8.65% |
|  |  | Diffusion mode | 2.36 | 0.56 | 12.78% | 2.61% | 7.58%, 18.41% |
|  |  | Topological proximity to maximal pathology sites | 3.05 | 0.48 | 4.70% | 1.32% | 0.59%, 8.00% |
|  | Functional connectivity | |  | | | | |
|  |  | Nodal strength | 1.71 | -0.26 | 2.26% | 1.01% | -0.87%, 5.34% |
|  |  | Diffusion mode | 1.49 | -0.40 | 5.00% | 1.27% | 0.71%, 9.71% |
|  |  | Topological proximity to maximal pathology sites | 3.14 | 0.58 | 10.17% | 1.37% | 1.93%, 16.41% |
|  | Cortical distance | |  | | | | |
|  |  | Nodal strength | 4.17 | -0.19 | 7.21% | 1.87% | -0.86%, 12.99% |
|  |  | Diffusion mode | 1.31 | -0.41 | 7.04% | 1.93% | 2.01%, 12.00% |
|  |  | Proximity to maximal pathology sites | 6.97 | 0.41 | 5.14% | 1.10% | 0.10%, 9.30% |
|  |  |  |  |  |  | | |
|  |  |  |  | Total R^2^ | 58.05% | 9.25% | 41.96%, 70.11% |
|  |  |  |  |  |  |  |  |
| Hypo-metabolism |  | |  | | | | |
|  | Structural connectivity | |  | | | | |
|  |  | Nodal strength | 2.88 | 0.14 | 1.76% | 1.03% | -0.75%, 4.26% |
|  |  | Diffusion mode | 3.84 | 0.37 | 9.94% | 1.95% | 3.39%, 16.21% |
|  |  | Topological proximity to maximal pathology sites | 2.46 | 0.04 | -0.26% | 1.07% | -0.96%, 0.42% |
|  | Functional connectivity | |  | | | | |
|  |  | Nodal strength | 1.34 | -0.35 | 10.19% | 1.79% | 3.88%, 16.51% |
|  |  | Diffusion mode | 1.62 | -0.29 | 4.75% | 1.13% | -0.34%, 9.67% |
|  |  | Topological proximity to maximal pathology sites | 1.43 | 0.36 | 21.10% | 3.33% | 11.35%, 30.19% |
|  | Cortical distance | |  | | | | |
|  |  | Nodal strength | 1.85 | -0.10 | 3.81% | 1.56% | -0.17%, 8.00% |
|  |  | Diffusion mode | 1.29 | -0.14 | 2.09% | 1.02% | -1.21%, 5.31% |
|  |  | Proximity to maximal pathology sites | 4.25 | 0.21 | 2.89% | 1.64% | -0.26%, 6.22% |
|  |  |  |  |  |  | | |
|  |  |  |  | Total R^2^ | 56.13% | 6.89% | 46.67%, 64,64% |
|  |  |  |  |  |  |  |  |
| Amyloid |  | |  | | | | |
|  | Structural connectivity | |  | | | | |
|  |  | Nodal strength | 2.99 | -0.25 | 6.16% | 0.85% | -0.50%, 12.48% |
|  |  | Diffusion mode | 2.91 | -0.20 | 2.03% | 0.42% | -0.78%, 4.63% |
|  |  | Topological proximity to maximal pathology sites | 8.30 | 0.11 | 1.37% | 0.16% | 0.47%, 2.24% |
|  | Functional connectivity | |  | | | | |
|  |  | Nodal strength | 1.28 | 0.27 | 6.23% | 0.75% | 0.24%, 11.67% |
|  |  | Diffusion mode | 1.53 | -0.02 | 1.69% | 0.39% | -0.79%, 4.17% |
|  |  | Topological proximity to maximal pathology sites | 1.44 | 0.54 | 24.18% | 1.38% | 15.07%, 33.64% |
|  | Cortical distance | |  | | | | |
|  |  | Nodal strength | 8.58 | 0.31 | 6.91% | 0.66% | 2.08%, 11.86% |
|  |  | Diffusion mode | 1.34 | 0.27 | 3.13% | 0.70% | -0.88%, 7.04% |
|  |  | Proximity to maximal pathology sites | 14.69 | 0.30 | 3.86% | 0.38% | 0.66%, 6.99% |
|  |  |  |  |  |  | | |
|  |  |  |  | Total R^2^ | 55.39% | 5.62% | 44,33%, 64.94% |
|  |  |  |  |  |  |  |  |
| Tau |  |  |  |  |  |  |  |
|  | Structural connectivity | |  | | | | |
|  |  | Nodal strength | 2.73 | 0.40 | 3.13% | 1.01% | 0.18%, 5.80% |
|  |  | Diffusion mode | 2.44 | 0.58 | 9.84% | 3.04% | 3.67%, 15.64% |
|  |  | Topological proximity to maximal pathology sites | 4.85 | 0.47 | 5.41% | 2.13% | 0.78%, 9.66% |
|  | Functional connectivity | |  | | | | |
|  |  | Nodal strength | 1.83 | -0.30 | 4.00% | 1.43% | -0.30%, 8.05% |
|  |  | Diffusion mode | 1.52 | -0.37 | 3.01% | 1.42% | -0.37%, 6.09% |
|  |  | Topological proximity to maximal pathology sites | 2.56 | 0.59 | 10.24% | 1.31% | 4.32%, 16.03% |
|  | Cortical distance | |  | | | | |
|  |  | Nodal strength | 5.80 | 0.25 | 1.50% | 0.88% | -1.80%, 4.65% |
|  |  | Diffusion mode | 1.29 | 0.06 | 0.13% | 0.78% | -1.26%, 1.55% |
|  |  | Proximity to maximal pathology sites | 10.66 | 0.64 | 11.66% | 1.58% | 3.15%, 19.34% |
|  |  |  |  |  |  | | |
|  |  |  |  | Total R^2^ | 48.71% | 8.82% | 37.04%, 58.62% |

VIF = Variance inflation factor; Pearson's r = Pearson correlation coefficient between the regressor and the regional biomarker estimated from the whole sample; LMG = Lindeman, Merenda, and Gold contribution; SD = standard deviation; 95% CI = 95% confidence interval. Mean and standard deviation of LMG contributions are obtained from the 1000 cross-validation test sets.

**Supplementary Figure 4. Biomarkers for APOE-ε4 carriers and non-carriers.**


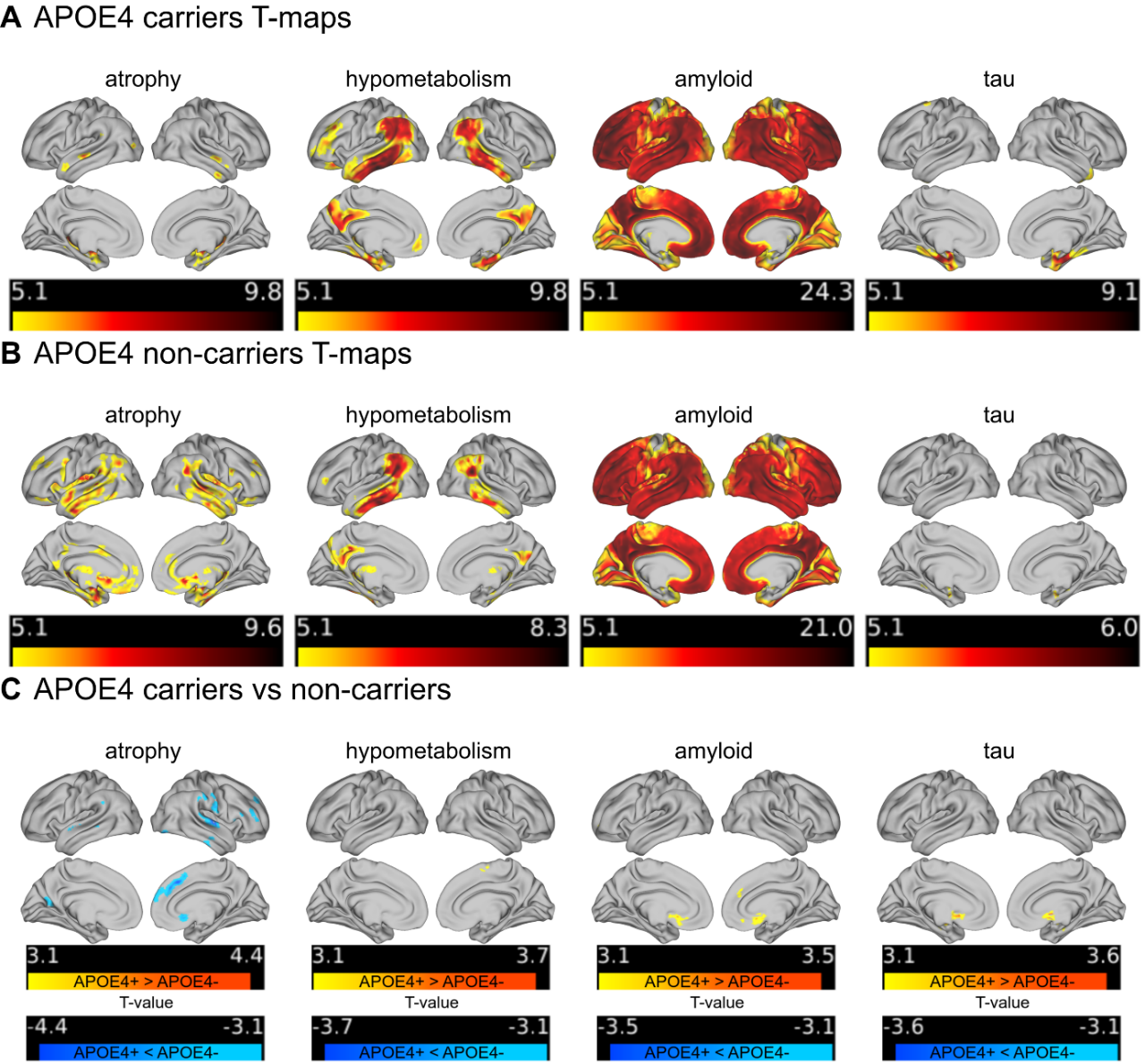


(**A**) and (**B**) Biomarkers T-maps for all APOE-ε4 non-carriers and matched carriers, thresholded at Bonferroni corrected p < 0.05 and cluster size k ≥ 50 voxels. (**C**) Significant differences between all APOE-ε4 non-carriers and matched carriers’ biomarkers, thresholded at p < 0.001 uncorrected and cluster size k ≥ 50 voxels, Welch’s t-test. N = 212 evenly distributed across the carrier and non-carrier groups, except for the tau-PET sample where n= 74. APOE4 = apolipoprotein E ε4.

**Supplementary Table 3. Identification of maximal pathology sites in APOE-ε4-status specific samples.**

| **Pathology** | **APOE-ε4 status** | **Global maximum location** | **Local maximum location** | **Side** | **MNI coordinates** | | | **Brainnetome region** | **Peak T** |
| --- | --- | --- | --- | --- | --- | --- | --- | --- | --- |
|  |  |  |  |  | **x** | **y** | **z** |  |  |
| Atrophy | pos | Amyg |  | R | 18 | -3 | -15 | mAmyg, medial amygdala | 7.2 |
|  |  | NAC |  | L | -26 | -14 | -10 | NAC, nucleus accumbens | 6.1 |
|  |  |  | cHipp | L | -28 | -39 | -3 | cHipp, caudal hippocampus | 5.5 |
|  |  |  |  |  |  |  |  |  |  |
|  | neg | rHipp |  | L | -28 | -10 | -15 | rHipp, rostral hippocampus | 6.4 |
|  |  |  | cHipp | L | -30 | -36 | -2 | cHipp, caudal hippocampus | 6 |
|  |  |  |  |  |  |  |  |  |  |
| Hypometabolism | pos | iTG |  | L | -56 | -28 | -27 | A20cv, caudoventral of area 20 | 8.1 |
|  |  |  | iTG | L | -57 | -48 | -12 | A20cl, caudolateral of area 20 | 7.7 |
|  |  |  | mTG | L | -60 | -27 | -12 | A21c, caudal area 21 | 6.4 |
|  |  |  | FuG | L | -42 | -66 | -15 | A37lv, lateroventral area 37 | 6.1 |
|  |  |  |  |  |  |  |  |  |  |
|  | neg | cHipp |  | L | -30 | -18 | -16 | cHipp, caudal hippocampus | 5.3 |
|  |  |  | Otha | L | -15 | -36 | 3 | Otha, occipital thalamus | 5.1 |
|  |  |  | cHipp | L | -33 | -26 | -12 | cHipp, caudal hippocampus | 4.7 |
|  |  |  | LinG | L | -28 | -46 | -3 | rLinG, rostral lingual gyrus | 4.2 |
|  |  | AG |  | R | 48 | -46 | 26 | A39rv, rostroventral area 39 (PGa) | 5.3 |
|  |  |  |  |  |  |  |  |  |  |
| Amyloid | pos | dlPFC |  | L | -44 | 40 | 10 | A9/46v, ventral area 9/46 | 17.5 |
|  |  |  | PCC | R | 3 | -64 | 36 | A31, area 31 (Lc1) | 17.5 |
|  |  |  | dPCC | L | -2 | -38 | 39 | A23d, dorsal area 23 | 17.4 |
|  |  |  | sOccG | L | -22 | -62 | 36 | lsOccG, lateral superior occipital gyrus | 17 |
|  |  |  |  |  |  |  |  |  |  |
|  | neg | mPOS |  | L | -6 | -56 | 10 | dmPOS, dorsomedial parietooccipital sulcus (PEr) | 15.4 |
|  |  |  | PCC | L | -2 | -54 | 32 | A31, area 31 (Lc1) | 14.7 |
|  |  |  | FuG | R | 54 | -60 | 0 | A37dl, dorsolateral area37 | 14 |
|  |  |  | vPCC | R | 9 | -52 | 8 | A23v, ventral area 23 | 13.7 |
|  |  |  |  |  |  |  |  |  |  |
| Tau | pos | mAmyg |  | R | 21 | -8 | -12 | mAmyg, medial amygdala | 10.7 |
|  |  |  | cHipp | R | 32 | -27 | -12 | cHipp, caudal hippocampus | 8.3 |
|  |  |  | EC | R | 22 | -12 | -30 | A28/34, area 28/34 (EC, entorhinal cortex) | 8.2 |
|  |  |  |  |  |  |  |  |  |  |
|  | neg | LinG |  | R | 4 | -90 | -10 | cLinG, caudal lingual gyrus | 5.5 |
|  |  | AG |  | R | 42 | -66 | 48 | A39rd, rostrodorsal area 39 (Hip3) | 5.1 |
|  |  |  | AG | R | 41 | -78 | 32 | A39c, caudal area 39 (PGp) | 4 |

We report first global maximum and up to three local maxima, and possibly following maxima if the number of bilateral significant regions is not the same for APOE-ε4-positive and negative groups. These maximal pathology regions were further used as proxies of epicenters.

pos = positive; neg = negative; R = right; L = left; Amyg = amygdala; AG = angular gyrus; cHipp = caudal hippocampus; rHipp = rostral hippocampus; dlPFC = dorsolateral prefrontal cortex; EC = entorhinal cortex; FuG = fusiform gyrus; iTJ = inferior temporal gyrus; mTG = middle temporal gyrus; LinG = lingual gyrus; MNI = Montreal national institute; NAC = nucleus accumbens; Otha = occipital thalamus; PCC = posterior cingulate cortex; dPCC = dorsal posterior cingulate cortex; sOccG = superior occipital gyrus; mPOS = medial parietooccipital sulcus; vPCC = ventral posterior cingulate cortex. Nucleus accumbens was not included in maximal pathology site as basal ganglia regions were discarded.
